# c-di-AMP Accumulation Regulates Growth, Metabolism, and Immunogenicity of *Mycobacterium smegmatis*

**DOI:** 10.3389/fmicb.2022.865045

**Published:** 2022-05-24

**Authors:** Huanhuan Ning, Xuan Liang, Yanling Xie, Lu Bai, Wei Zhang, Lifei Wang, Jian Kang, Yanzhi Lu, Yanling Ma, Guangchun Bai, Yinlan Bai

**Affiliations:** ^1^Department of Microbiology and Pathogen Biology, Air Force Medical University, Xi’an, China; ^2^College of Life Sciences, Northwest University, Xi’an, China; ^3^School of Life Sciences, Yan’an University, Yan’an, China; ^4^Department of Pediatrics, Tangdu Hospital, Air Force Medical University, Xi’an, China; ^5^Graduate School, Chang’an University, Xi’an, China; ^6^Department of Immunology and Microbial Disease, Albany Medical College, Albany, NY, United States

**Keywords:** *Mycobacterium smegmatis*, *M. tuberculosis*, c-di-AMP, physiology, immunogenicity

## Abstract

Cyclic dimeric adenosine monophosphate (c-di-AMP) is a ubiquitous second messenger of bacteria involved in diverse physiological processes as well as host immune responses. *MSMEG_2630* is a c-di-AMP phosphodiesterase (*cnpB*) of *Mycobacterium smegmatis*, which is homologous to *Mycobacterium tuberculosis Rv2837c*. In this study, *cnpB*-deleted (Δ*cnpB*), -complemented (Δ*cnpB*::C), and -overexpressed (Δ*cnpB*::O) strains of *M. smegmatis* were constructed to investigate the role of c-di-AMP in regulating mycobacterial physiology and immunogenicity. This study provides more precise evidence that elevated c-di-AMP level resulted in smaller colonies, shorter bacteria length, impaired growth, and inhibition of potassium transporter in *M. smegmatis*. This is the first study to report that elevated c-di-AMP level could inhibit biofilm formation and induce porphyrin accumulation in *M. smegmatis* by regulating associated gene expressions, which may have effects on drug resistance and virulence of mycobacterium. Moreover, the *cnpB*-deleted strain with an elevated c-di-AMP level could induce enhanced Th1 immune responses after *M. tuberculosis* infection. Further, the pathological changes and the bacteria burden in Δ*cnpB* group were comparable with the wild-type *M. smegmatis* group against *M. tuberculosis* venous infection in the mouse model. Our findings enhanced the understanding of the physiological role of c-di-AMP in mycobacterium, and *M. smegmatis cnpB*-deleted strain with elevated c-di-AMP level showed the potential for a vaccine against tuberculosis.

## Introduction

*Mycobacterium tuberculosis* is the causative agent of tuberculosis (TB), which is a leading cause of infectious morbidity and mortality with 9.9 million new cases and 1.3–1.5 million deaths in 2020 (WHO, 2021). Currently, the TB burden remains high, mainly due to TB/HIV co-infection and the emergence of drug-resistant *M. tuberculosis* strains. Live-attenuated Bacille Calmette-Gue’rin (BCG) is the only licensed vaccine against TB. However, the efficiency of BCG varies greatly in protecting against adult pulmonary TB (Mangtani et al., 2014). Deeper insight into the mechanisms regulating physiological and immunological properties of *M. tuberculosis* is essential for the development of therapeutics and novel or alternative vaccines against TB.

Cyclic di-adenosine monophosphate (c-di-AMP) is a ubiquitous second messenger produced by bacteria and archaea including *Bacillus subtilis*, *Listeria monocytogenes*, *Streptococcus pneumoniae*, and *M. tuberculosis* (Bai and Bai, 2020; Yin et al., 2020), which regulates diverse cellular processes including bacterial growth, biofilm formation, potassium transport, and virulence (Yin et al., 2020). In bacteria, the c-di-AMP level is controlled by diadenylate cyclase (DisA or DAC) and phosphodiesterase (PDE) (He et al., 2020; Stulke and Kruger, 2020; Zarrella and Bai, 2020). In *M. tuberculosis*, Rv3586 (DisA) is the sole diadenylate cyclase (Bai et al., 2012), and Rv2837c is a cyclic nucleotide phosphodiesterase (CnpB) for c-di-AMP hydrolysis (Yang et al., 2014). Deletion of *cnpB* or overexpression of *disA* could increase c-di-AMP level in *M. tuberculosis* (Yang et al., 2014; He et al., 2016; Dey et al., 2017), BCG (Zhang et al., 2018; Ning et al., 2019), and *M. smegmatis* (Tang et al., 2015). It has been proved that c-di-AMP regulates bacterial length in *M. tuberculosis* and BCG (Yang et al., 2014; Ning et al., 2019), colony morphology in *M. smegmatis* (Zhang et al., 2013; Tang et al., 2015), potassium transport in *S. pneumoniae* (Bai et al., 2014), and virulence of *M. tuberculosis* (Yang et al., 2014; Dey et al., 2017). Furthermore, CnpB degrades nanoRNAs (RNA oligos of ≤ 5 nucleotides) (Postic et al., 2012) and even hydrolyzes c-di-GMP at a lower rate than it dose on c-di-AMP (He et al., 2016). In fact, more phenotypes including those mentioned above have been found in studies of other bacteria such as biofilm, envelope stress, antibiotic resistance, osmoregulation, and virulence (Corrigan et al., 2011; Whiteley et al., 2017; Hu et al., 2020). Thus, it is deduced that more phenotypes controlled by c-di-AMP still need to be discovered in mycobacteria.

Now, c-di-AMP has been considered a key mycobacterial pathogen-associated molecular pattern (PAMP) inducing host innate immune responses, including type I IFN response and autophagy (Woodward et al., 2010; Dey et al., 2015; Devaux et al., 2018; Ning et al., 2019; Singh et al., 2022). Additionally, c-di-AMP as an adjuvant enhanced antigen-induced Th1/Th2/Th17 pattern responses (Ebensen et al., 2011; Ning et al., 2021). It has been noted that *cnpB*-deleted strain of *M. tuberculosis* triggered a potent type I IFN response, showing the reduction of bacillary burden in the mouse model, implying virulence attenuation in *M. tuberculosis* (Yang et al., 2014; Dey et al., 2017). The adaptive immune responses play a major role in the elimination of intracellular mycobacteria. Previous studies showed that *disA*-overexpressing BCG with extra c-di-AMP as an endogenous adjuvant could induce enhanced immune responses after *M. tuberculosis* infection in mice (Ning et al., 2019) and provided improved protection compared with BCG against aerosol infection of *M. tuberculosis* in guinea pig (Dey et al., 2020). Therefore, studying the adaptive immune response induced by recombinant mycobacterium with an elevated c-di-AMP level may provide new methods for the improvement of the tuberculosis vaccine.

*Mycobacterium smegmatis* is a fast-grown and non-pathogenic mycobacterium and is frequently used as a genetic surrogate to study the physiological processes and gene function of other extremely slow-growing species such as *M. tuberculosis* (Maarsingh et al., 2019; T et al., 2020). *M. smegmatis* is also a vaccine vector for a live vaccine against TB. Several recombinant *M. smegmatis* with different antigens could induce protective immune responses in mice against tuberculosis (Junqueira-Kipnis et al., 2013; Alves Da Silva et al., 2014; Wang et al., 2014; Liu et al., 2015). *MSMEG_2630* is homologous with c-di-AMP phosphodiesterase *cnpB* of *M. tuberculosis* (Tang et al., 2015). In this study, *cnpB*-deleted (Δ*cnpB*), -complemented (Δ*cnpB*::C), and -overexpressed (Δ*cnpB*::O) strains of *M. smegmatis* were constructed to investigate the role of c-di-AMP in regulating mycobacterial physiology and immunogenicity. This study provided more evidence to clarify the role of c-di-AMP on bacterial physiology and immunogenicity and suggested the applicability of Δ*cnpB* for a vaccine against TB.

## Materials and Methods

### Bacterial Strains, Plasmids, Antibodies, and Animals

*Mycobacterium smegmatis* mc^2^-155 and *M. tuberculosis* H37Ra were obtained from the National Institutes for Food and Drug Control of China. Mycobacteria were grown in Middlebrook 7H9 broth (BD Difco) supplemented with 0.5% glycerol, 0.05% Tween 80, and 10% oleic acid-albumin-dextrose-catalase (OADC) (BD Difco) at 37°C. *E. coli* DH5α was grown in the LB medium at 37°C. When required, antibiotics were added as follows: zeocin 100 μg/ml, kanamycin 25 μg/ml, or hygromycin 150 μg/ml. Mycobacteria were plated on Middlebrook 7H10 agar (BD Difco) supplemented with 10% OADC for colony-forming unit (CFU) determination and were grown at 37°C. Recombinant purified proteins of CnpB (MSMEG_2630), DisA (MSMEG_6080), Ag85 (MSMEG_6398), and pyridoxamine 5′-phosphate oxidase (PdxH, MSMEG_5675) and their polyclonal antibody sera were prepared in our laboratory. The strains and plasmids used in this study are listed in Supplementary Table 1. Female BALB/c mice aged 6–8 weeks were purchased from the Animal Center of Air Force Medical University.

### Construction and Complementation of Mutants

*Mycobacterium smegmatis* Δ*cnpB* mutant was constructed by homologous recombination using a previously reported approach (Van Kessel and Hatfull, 2007). Briefly, upstream and downstream homologous fragments of *cnpB* were amplified by polymerase chain reaction (PCR) with *M. smegmatis* mc^2^-155 genomic DNA as the template. The two flanking DNA fragments were sequentially subcloned into pMSG360zeo at *Eco*RV/*Hin*dIII and *Xba*I/*Kpn*I restriction sites. The linear recombineering substrates flanked by homologous arms (*loxp*-*zeo*-*loxp*) were generated by *Eco*RV and *Kpn*I digestion. The linear DNA substrates were electroporated into *M. smegmatis* cells that carried plasmid of pJV53. Then, cells were plated on 7H10 agar containing 100 μg/ml zeocin. The recombinant clone was verified by PCR and western blot analysis. The plasmid of pJV53 expressing gp60/gp61 was lost spontaneously. Primers used in this study are listed in Supplementary Table 2.

To complement the mutant, the *cnpB* open reading frame (ORF) was cloned into the single-copy expression vector of PW51 between *Kpn*I/*Bam*HI sites, and this plasmid of PW51-*cnpB* was transformed into Δ*cnpB* to complement the mutation (Δ*cnpB*::C). Meanwhile, *cnpB* ORF was cloned into the multiple-copy shuttle expression vector of PW54 at *Hin*dIII site. The recombinant plasmid of PW54-*cnpB* was transformed into Δ*cnpB* to generate *cnpB*-overexpressed strain (Δ*cnpB*::O). Transformants were verified by PCR and western blot analysis.

### Western Blot Analysis

*Mycobacterium smegmatis* strains were grown in 15 ml of 7H9 broth supplemented with 0.5% glycerol, 0.05% Tween 80, and 10% OADC at 37°C until OD_600_ = 1.0. Bacteria were harvested and washed three times with phosphate-buffered saline (PBS). Pellets were resuspended in precooled PBS supplemented with proteinase inhibitor cocktail (Roche) and then lysed by MINI-BEAD BEATER (BioSpec). The lysates were centrifuged at 12,000 rpm for 20 min at 4°C, and the supernatants were collected. Besides, the bacterial culture supernatants were concentrated by vacuum freeze-drying for western blot analysis. The concentrations of proteins were measured by the bicinchoninic acid assay (BCA). Phenotypes of *M. smegmatis* strains were confirmed by western blot analysis with polyclonal antibodies as the primary antibody, as shown in Supplementary Table 1. PdxH was used as a loading control, and recombinant proteins were used as positive controls.

### Determination of c-di-AMP Concentration by High-Performance Liquid Chromatography

*Mycobacterium smegmatis* strains were grown to OD_600_ = 1.0 in 7H9 broth with 10% OADC. Intracellular nucleotides were extracted from bacterial pellets according to the reported method (Burhenne and Kaever, 2013). Briefly, bacterial pellets were resuspended in extraction buffer (acetonitrile: methanol: water = 2:2:1), incubated on ice, boiled at 95°C for 10 min, and incubated on ice. The lysates were centrifuged for 10 min at 20,800 × *g* at 4°C. Supernatants were transferred to a new tube. The extraction procedure was repeated two times. The pooled extractions were lyophilized and dissolved in ddH_2_O. c-di-AMP levels were determined by high-performance liquid chromatography (HPLC) as previously reported (Ning et al., 2019). Chemically synthesized c-di-AMP (InvivoGen) was used as a standard substance to generate a standard curve. The concentrations of c-di-AMP in *M. smegmatis* strains (1 g wet weight) were displayed as nanomoles per liter (nM).

### Determination of Bacterial Growth and Size

*Mycobacterium smegmatis* strains (2 × 10^5^CFU) were grown in 15 ml 7H9 broth with 10% OADC or in Sauton’s medium in 50 ml tube under shaking (80 rpm and 180 rpm) or stationary conditions at 37°C. The growth of each strain was determined by measuring OD_600_ at indicated time points. The bacterial length was determined according to our previous study (Ning et al., 2019). Briefly, bacteria in the late-log phase were collected, washed with PBS, and resuspended in PBS. Bacterial suspensions were dropped on copper grids (200 mesh) for 10 min incubation, and excess bacterial suspension was removed by the filter paper. Bacteria were stained with phosphotungstic acid, and the bacteria were observed by transmission electron microscopy (TEM) (TECNAIG2 Spirit Biotwin). The bacterial length was measured by ImageJ software. The individual cells were manually traced in ImageJ and converted bacterial arbitrary units to actual size according to the scale of 5 μm. Bacterial cells in clumps were omitted during measurement.

### Biofilm Formation and Biomass Quantification

*Mycobacterium smegmatis* strains were grown in a 7H9 medium with 10% OADC to OD_600_ = 0.8–1.0 at 37°C. Bacteria pellets were resuspended and washed two times by Sauton’s medium in the absence of Tween 80. The cultures were diluted by Sauton’s medium at OD_600_ = 0.2 and inoculated into 24-well (2 ml/well, for imaging) or 96-well (0.2 ml/well, for quantification) plates. The plates were incubated statically at 37°C for 3–5 days. Biomass was quantified by a crystal violet assay. Briefly, biofilms in the well were washed with ddH_2_O three times to remove the medium. The biofilms were dried at 37°C and incubated with 200 μl of 1% (w/v) crystal violet for 30 min and washed two times with ddH_2_O. About 200 μl of 95% ethanol was added to dissolve the biofilm. The OD_570_ was recorded by a microplate reader. Each experiment was performed in a set of three replicates.

### Flow Cytometry Analysis of Long-Term Cultured Bacteria

*Mycobacterium smegmatis* strains were inoculated in a 7H9 medium and cultured for 3 days (stationary phase) at 80 rpm at 37°C. Subsequently, the cultures were transferred to a 37°C incubator for static culture avoiding light. After incubation at 14 and 60 weeks, bacteria were harvested and washed with PBS. Red autofluorescence of the bacterial cells was monitored by the flow cytometer (BeckmanCoulter EXPO32) in the FL3 channel (excitation 488 nm with a 610LP emission filter). Fresh bacteria (3 days) were used as the control. Flow cytometry data were analyzed using the FlowJo V10.

### Absorption and Fluorescence Spectra of Long-Term Cultured Bacterial Supernatant

The 14- and 60-week supernatants of *M. smegmatis* strains were collected and filtered with a 0.22 μm filter membrane. Absorption spectra of supernatants were recorded by a multifunctional microplate reader (Tecan Spark) from 100 to 1,000 nm. Then, fluorescence measurements were taken with TECAN Spark at the excitation wavelength (λ_excitation_) of 400 nm.

### RNA-seq and qRT-PCR Analysis

Total RNAs were extracted from *M. smegmatis* by TRIzol reagent (Invitrogen) according to the manufacturer’s protocol. RNA quality was assessed on an Agilent 2100 Bioanalyzer (Agilent Technologies) and RNase-free agarose gel electrophoresis. mRNA was enriched by removing rRNA by Ribo-Zero Magnetic Kit (Epicenter) for library construction. RNA sequencing (RNA-seq) was performed using Illumina Novaseq6000 by Gene Denovo Biotechnology Co., Ltd., (Guangzhou, China). The expression levels of differential genes were further verified by real-time quantitative PCR (qRT-PCR). In total, 500 ng total RNA was reverse-transcribed using the HiScript III cDNA Synthesis Kit (Vazyme). The transcriptional levels of target genes were determined by qRT-PCR using SYRB Master Mix (Vazyme). Primers used in the qRT-PCR assay are listed in Supplementary Table 2. The relative transcriptional levels of genes were presented as 2^–Δ^ ^Δ^ ^Ct^ with *M. smegmatis sigA* as a reference gene.

### Mice Vaccination and Challenge With *M. tuberculosis*

Female BALB/c mice were anesthetized with an intraperitoneal injection of 50 mg/kg pentobarbital sodium. Mice were immunized with 10^7^CFU *M. smegmatis* in 100 μl PBS for three times at 2-week intervals through a subcutaneous (s.c) route on the back. An equal volume of PBS was injected into control mice. Then, 4 weeks after immunization, mice were challenged intravenously (i.v) with 5 × 10^4^ CFU *M. tuberculosis* H37Ra in 200 μl PBS.

### ELISA

Whole blood was collected at 4 weeks post-immunization and 8 weeks post-infection. The whole blood was kept at 37°C for 30 min to collect the supernatant as serum. Plates were coated with *M. smegmatis* total protein extract of mycobacteria and CnpB. The serum was diluted at 1:200 with PBS. Antigen-specific IgG subclasses were determined by indirect enzyme-linked immunosorbent assay (ELISA). For cytokine measurement of IFN-γ, IL-2, and IL-10, splenocyte supernatants were collected after 72 h stimulation with *M. smegmatis* protein extract (25 μg/ml) as described in our previous study (Ning et al., 2019). Double antibody sandwich ELISA was performed according to the manufacturer’s instructions (Thermo Fisher Scientific).

### Splenocytes Proliferation Assay

Spleen single-cell suspensions were prepared as we previously described (Ning et al., 2019). For cells proliferation assay, 1 × 10^6^ cells were seeded in 96-well microplates and stimulated with CnpB (5 μg/ml), *M. smegmatis* protein extract (25 μg/ml), or *M. tuberculosis* H37Ra protein extract (25 μg/ml) as indicated. After stimulation at 37°C for 68 h with 5% CO_2_, MTS reagents (Promega) were added in each well and plates were incubated for another 4 h at 37°C. The absorbance at 490 nm was recorded using a microplate reader. The stimulated index was calculated as described in our previous study (Lu et al., 2018).

### Histopathology and CFU Enumeration

For histopathology, the upper lobes of the left lungs were fixed in 10% buffered formalin for 24 h. Then, sections of 5 μm in thickness of tissues were cut into glass slides. Hematoxylin–eosin (H&E) staining for pathohistological analysis was performed by the Department of Histopathology (Air Force Medical University, China). Sections were observed under a light microscope (Olympus). At 8 weeks after *M. tuberculosis* infection, the lung and the spleen were removed and homogenized in sterile PBS. Homogenates were diluted and spread on 7H10 agar plates with 10% OADC enrichment. Plates were then incubated at 37°C for 3 weeks. The data are represented in Log_10_CFU (lgCFU).

### Ethics Statement

Animal experiments were performed according to the approval and guidance of the Institutional Animal Ethics Center of the Air Force Medical University, and the approval number is 20190213.

### Statistical Analysis

Statistical analysis was performed by GraphPad Prism 8.0 software. Statistical significance was determined by Student’s *t*-test or for multiple comparisons by ANOVA. *P* < 0.05 were considered statistically significant. Data are shown as means ± SEM or as individual data points, i.e., “*” denotes *p* < 0.05, “^**^” denotes *p* < 0.01, “^***^” denotes *p* < 0.001, and “^****^” denotes *p* < 0.0001.

## Results

### Deletion of CnpB Increases c-di-AMP Level in *M. smegmatis*

**FIGURE 1:**
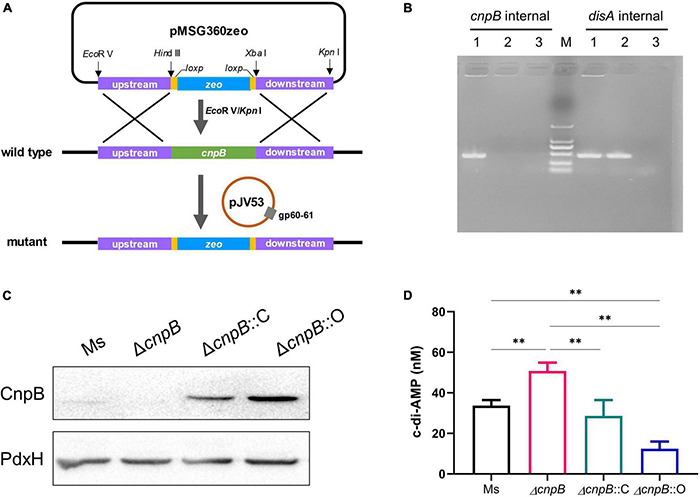
Deletion of *cnpB* led to elevated bacterial c-di-AMP level. **(A)** Schematic diagram showing the *cnpB* knockout strategy in *M. smegmatis*. **(B)** PCR verification for the deletion of *cnpB* in *M. smegmatis*. *disA* (*MSMEG_6080*) was used as a reference gene. The templates were wild-type strain of *M. smegmatis* (lane 1), *cnpB* mutant (lane 2), and negative control without template (lane 3), respectively. **(C)** CnpB expression was determined using western blot with bacterial lysates of wild-type (*M. smegmatis*, Ms), *cnpB* mutant (Δ*cnpB*), *cnpB*-complemented strain in Δ*cnpB* (Δ*cnpB*::C), and *cnpB*-overexpressed strain in Δ*cnpB* (Δ*cnpB*::O), respectively. Pyridoxamine 5′-phosphate oxidase (PdxH, MSMEG_5675) was used as a loading control. **(D)** The levels of c-di-AMP in each strain were assayed by HPLC. ^**^p < 0.01.

*Mycobacterium smegmatis* CnpB shared 73% identity (247/338 aa) with *M. tuberculosis* CnpB (Supplementary Figure 1A). In addition, *M. smegmatis* CnpB carries a highly conserved motif of Asp-His-His (DHH) (Supplementary Figure 1B), which is indispensable for the degradation of c-di-AMP (Yang et al., 2014; Tang et al., 2015). In this study, *cnpB* mutant (Δ*cnpB*) of *M. smegmatis* mc^2^155 was generated by homologous recombination through Che9c gp60/gp61 recombinase-assisted genome engineering (Figures 1A,B). Meanwhile, complemented (Δ*cnpB*::C) and overexpressed (Δ*cnpB*::O) strains of Δ*cnpB* were generated by different plasmids with the *cnpB* gene from *M. smegmatis*. These strains were verified by PCR and western blot analysis (Figures 1B,C). CnpB expression was restored in Δ*cnpB*::C and Δ*cnpB*::O (Figure 1C). High-performance liquid chromatography (HPLC) analysis showed that intracellular c-di-AMP concentration was elevated 1.5-folds in Δ*cnpB* than that of wild-type strain (*p* < 0.01) (Figure 1D). The level of c-di-AMP in Δ*cnpB*::C was lower than that of Δ*cnpB* but was comparable to that of wild-type (Figure 1D). However, the c-di-AMP level of Δ*cnpB*::O was about a quarter of that in Δ*cnpB* (Figure 1D), which could be explained that more c-di-AMP was hydrolyzed in *M. smegmatis cnpB*-overexpressing strain. These results showed that the c-di-AMP level of *M. smegmatis* could be controlled by the expression level of CnpB.

### c-di-AMP Controls the Colony Morphology of *M. smegmatis*

**FIGURE 2:**
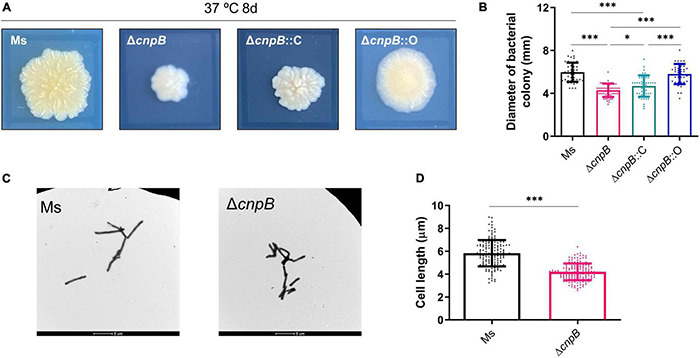
Determination of bacterial colony morphologies and bacterial lengths. **(A)** Colony morphologies on 7H10 + OADC plates after 8-day culture at 37°C. The black rectangle around each colony is 10 mm × 10 mm. **(B)** The measurement of colony diameter in panel **(A)** (40–50 colonies). **(C)** Observation of the indicated bacterial strains with a transmission electron microscope after culturing in 7H9 + OADC medium for 3 days. **(D)** The length of bacterial cells in panel **(C)** was measured (about 150 bacteria). *p < 0.05, ^***^p < 0.001.

It has been found that increased c-di-AMP level by DisA overexpression in *M. smegmatis* led to abnormal phenotypes, including cell expansion, cell bulge phenotype, bacterial aggregation, and loss of motility (Zhang and He, 2013; Tang et al., 2015). It was reported that the morphology of CnpB-overexpressed strain was normal as wild-type *M. smegmatis* (Tang et al., 2015). In our study, the Δ*cnpB* colonies showed a smaller, smooth, and moist phenotype compared with that of the wild-type (Figure 2A). The colonies of Δ*cnpB*::O showed a similar appearance but has fewer wrinkles (Figure 2A). Colony diameter measurements showed that Δ*cnpB* formed smaller colonies with 28% reduction in diameter (*p* < 0.001) (Figures 2A,B). Δ*cnpB*::C just partially restored the colony size compared with Δ*cnpB* (*p* < 0.05) and smaller than wild-type (*p* < 0.001) (Figures 2A,B). However, Δ*cnpB*::O with the lowest c-di-AMP level exhibited a comparable colony size with wild-type, but other appearances seemed to be different from wild-type (Figures 2A,B).

Elevated c-di-AMP level in *M. tuberculosis* by CnpB deletion or in BCG by DisA overexpression led to a reduction of the single bacterial length (Yang et al., 2014; Ning et al., 2019). We speculated that the smaller bacterial colony is due to the smaller bacterial length. As expected, transmission electron microscope (TEM) observation showed that Δ*cnpB* (4.16 ± 1.73 μm) exhibited a bacterial length reduction of approximately 28% relative to the wild-type (5.83 ± 1.15 μm) *M. smegmatis* (Figures 2C,D). These results confirmed previous reports that the c-di-AMP level affected colony morphologies as well as bacterial length in mycobacteria and suggested that other phenotypes were controlled by c-di-AMP.

### c-di-AMP Affects *M. smegmatis* Growth

**FIGURE 3:**
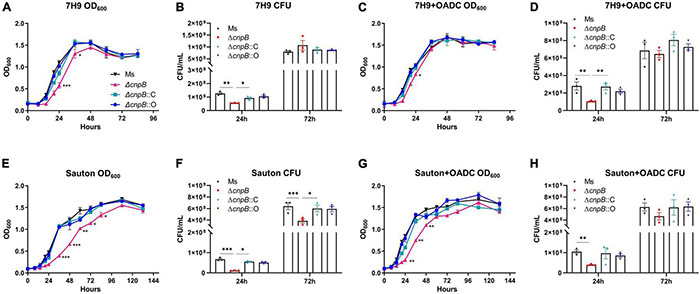
Detection of bacteria growth in liquid media (180 rpm). Each strain was inoculated at 2.5 × 10^6^CFU/ml in media of 7H9 **(A)**, 7H9 + OADC **(C)**, Sauton’s **(E)**, and Sauton’s + OADC **(G)**. Bacteria were monitored at OD_600_ **(A,C,E,G)** and corresponding CFUs **(B,D,F,H)** were numbered at indicated time points, respectively. The growth curves were drawn with the results of three independent experiments with three duplicate cultures. *p < 0.05, ^**^p < 0.01, ^***^p < 0.001.

In *M. tuberculosis*, deletion of c-di-AMP phosphodiesterase did not affect *M. tuberculosis* growth by OD_600_ (Yang et al., 2014), and a similar result was observed in *L. monocytogenes* (Witte et al., 2013). The growth of wild-type, Δ*cnpB*, Δ*cnpB*::C, and Δ*cnpB*::O was monitored in Middlebrook 7H9 medium and Sauton’s medium. Our data showed that the growth rate of Δ*cnpB* was slower in the exponential phase in 7H9 determined at both OD_600_ and CFU detections but reached similar levels at the stationary phase (Figures 3A–D). Meanwhile, Δ*cnpB*::C and Δ*cnpB*::O had similar growth rates as that of wild-type in 7H9 broth without the addition of OADC (Figures 3A,B). Similar results were observed when bacteria were grown in 7H9 + OADC (Figures 3C,D). Sauton’s medium is a minimal medium that does not contain exogenous proteins and is commonly used for the preparation of mycobacterial culture filtrates (Venkataswamy et al., 2012). In Sauton’s medium with or without OADC enrichment, Δ*cnpB* had a significantly reduced growth rate (Figures 3E–H). Both Δ*cnpB*::C and Δ*cnpB*::O showed a similar growth rates/CFUs to that of wild-type in 7H9 and Sauton’s medium with OADC (Figures 3E–H). The reduction of Δ*cnpB* growth was also found under slow shaking conditions (80 rpm) (Supplementary Figures 2A–H). Collectively, elevated c-di-AMP level impaired *M. smegmatis* growth, especially in media with less complex nutrients.

### Increasing c-di-AMP Level Causes a Reduction in Biofilm Formation in *M. smegmatis*

**FIGURE 4:**
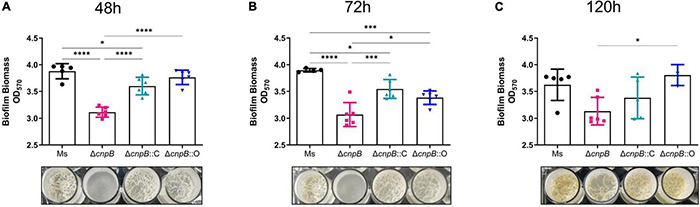
Biofilm formation and biomass quantification. Biomass was quantified by a crystal violet assay at 48 **(A)**, 72 **(B)**, and 120 h **(C)**. The corresponding biofilm images for each strain are shown below each histogram. *p < 0.05, ^***^p < 0.001, ^****^p < 0.0001.

It has been reported that smooth or rough/wrinkled colony morphology was associated with the biofilm formation in several bacteria (Friedman and Kolter, 2004; Uhlich et al., 2006; Bharti et al., 2020). Further, the ability of Δ*cnpB* strain to form biofilm was determined in Sauton’s medium at 48, 72, and 120 h respectively, and biomass was quantified by the crystal violet assay. It was observed that Δ*cnpB* could not form the biofilm with rough surfaces like *M. smegmatis* and showed a smooth pellicle with low biomass quantifications by crystal violet assay in 48 and 72 h culture (Figures 4A,B). Though Δ*cnpB*::C showed a decrease in biofilm formation in 72 h culture, but more biomass quantification was detected in 120 h culture than Δ*cnpB* and was comparable to the wild-type strain (Figure 4C). To our surprise, Δ*cnpB*::O with decreased c-di-AMP level showed similar biofilm formation as Δ*cnpB*::C (Figures 4A–C). Nevertheless, our experimental results demonstrated that c-di-AMP could regulate the biofilm formation of mycobacterium, so the mechanisms related to biofilm regulation should be investigated further.

### c-di-AMP Regulates Porphyrin Metabolism in the Long-Term Culture of *M. smegmatis*

**FIGURE 5:**
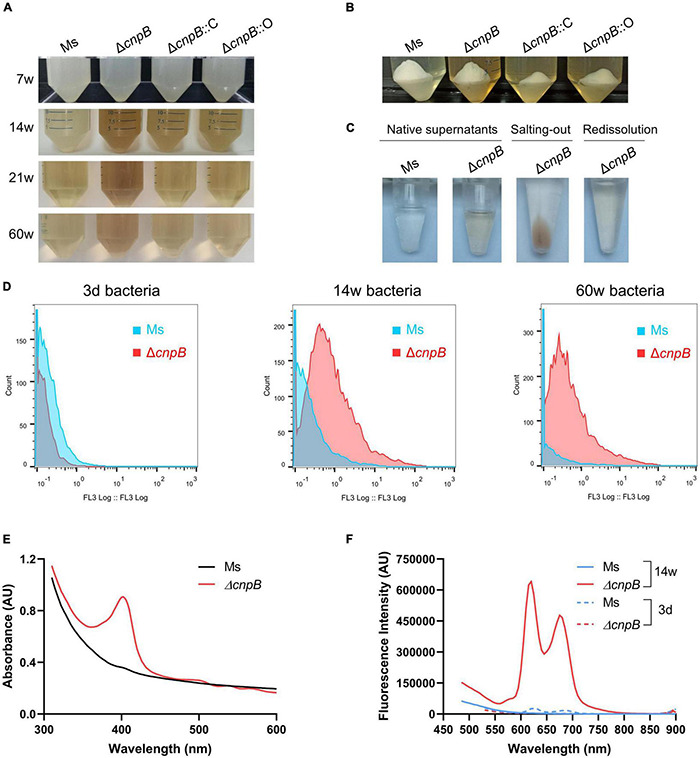
Pigment produced by Δ*cnpB* in long-term stationary culture. **(A)** The appearance of each strain was cultured statically for 7, 14, 21 and 60 weeks. Images were captured after gentle mixing. **(B)** At 14-week incubation, the bacterial pellets and supernatants were separated by centrifugation. **(C)** Observation of wild-type and Δ*cnpB* supernatants at 14 weeks. Next, Δ*cnpB* supernatant was salted-out by saturated ammonium sulfate [(NH_4_)_2_SO_4_] and protein was collected by centrifugation. Then, protein precipitation by salting-out was redissolved by PBS. **(D)** Autofluorescence of 3 days, 14 weeks, and 60 weeks of bacterial cells were monitored by the flow cytometer (FL3, excitation 488 nm with a 610LP emission filter). **(E)** Absorption spectra of 14-week supernatants were recorded by full-wavelength (100–1,000 nm) scanning. **(F)** Fluorescence measurements of 14-week supernatants were analyzed at the excitation wavelength (λ_excitation_) 400 nm.

Unexpectedly, we observed brown coloration in the culture of Δ*cnpB*, Δ*cnpB*::C, and Δ*cnpB*::O over 14 weeks, and supernatants of Δ*cnpB*::C and Δ*cnpB*::O showed lighter brown color than that of Δ*cnpB* (Figures 5A,B). The brown coloration of Δ*cnpB*::C and Δ*cnpB*::O supernatants gradually decreases with incubation time and were comparable to wild-type of *M. smegmatis* in 60-week culture (Figure 5A). After centrifuging, the brown coloration was retained in supernatant rather than bacterial pellets of *M. smegmatis* strains, suggesting that the unknown brown material substance was water soluble (Figure 5B). In addition, the brown pigment could not be removed by dialysis but be salted out from Δ*cnpB* supernatant by saturated (NH_4_)_2_SO_4_, which suggested that the brown pigment could bind to protein (Figure 5C and Supplementary Figure 3A). According to the flow cytometry experiments, Δ*cnpB* cells from long-term (14 and 60 weeks) rather than fresh (3 days) culture revealed red fluorescence (Figure 5D). Then, an absorbance peak at 400 nm was found in Δ*cnpB* supernatant of 14-week culture recorded by full-wavelength (100–1,000 nm) scanning (Figure 5E) and a lower absorbance peak in that of Δ*cnpB*::C and Δ*cnpB*::O (Supplementary Figure 3B). It was observed that *M. smegmatis* produced a dark brown fluorescent pigment in long-term culture, which was identified as porphyrin by mass spectrometry analysis and ^1^H-NMR spectra (Nikitushkin et al., 2016). Porphyrin has the typical fluorescence at 400 nm and fluorescence emission maxima at 625 and 675 nm (excitation wavelength, 400 nm) (Gouterman, 1959, 1961; Dolphin, 1978; Nikitushkin et al., 2016). Further, we found the characteristic fluorescence of Δ*cnpB* supernatant at 625 and 675 nm with an excitation wavelength of 400 nm (Figure 5F and Supplementary Figure 3C). Taken together, our results suggested that the elevated c-di-AMP level may increase porphyrin production in *M. smegmatis*.

### c-di-AMP Modulates Broad Gene Expression of *M. smegmatis* Genes

**FIGURE 6:**
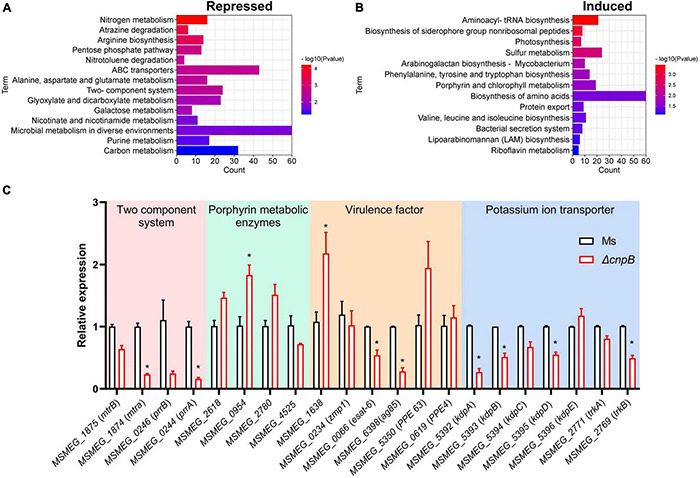
Transcriptional profiles and genes expression of Δ*cnpB*. Strains were inoculated in 7H9 + OADC medium and cultivated to the late logarithmic stage (3 d) and bacteria pellets were collected for RNA extraction. Differentially expressed genes (DEGs) were screened by RNA-seq with both false discovery rate (FDR) < 0.05 and | log2FC| > 1. KEGG pathway enrichment is associated with DEGs that are repressed **(A)** or induced **(B)** in Δ*cnpB* compared with wild-type *M. smegmatis* strain. Count means the number of differential gene reads enriched to indicated terms **(A,B)**. **(C)** The transcription levels of indicated genes were verified by real-time quantitative PCR. *p < 0.05.

To further explore gene expression profiles regulated by c-di-AMP, *M. smegmatis* and Δ*cnpB* pellets of late logarithmic phase cultures were harvested for RNA-seq analysis. Differentially expressed genes (DEGs) were analyzed at the cutoff of both false discovery rate (FDR) < 0.05 and | log2FC| > 1. Overall, it was found that a total of 2,828 genes were differentially expressed between Δ*cnpB* and wild-type, including 1,870 upregulated and 958 downregulated compared with wild-type strain. Kyoto Encyclopedia of Genes and Genomes (KEGG) pathway enrichment showed that several major metabolism-related genes were repressed in Δ*cnpB*, including nitrogen, alanine, aspartate, and glutamate, microbial metabolism in diverse environments, galactose metabolism, purine, carbon, and so on (Figure 6A). On the other side, elevated c-di-AMP in *M. smegmatis* induced expressions of genes involved in the biosynthesis of aminoacyl-tRNA, amino acids and so on (Figure 6B). We also noticed that gene expressions of porphyrin and chlorophyll metabolism were also induced in Δ*cnpB* (Figure 6B).

Further, qRT-PCR was used for the analysis of gene expressions associated with the phenotypes found above. We had observed that Δ*cnpB* with high c-di-AMP reduced the biofilm formation in the 7H9 complete medium (Figure 4). The impaired or absent function of the two-component system results in defective biofilm formation in *M. smegmatis* (Nguyen et al., 2010; Li et al., 2020). Deletion of *cnpB* with increased c-di-AMP downregulated the genes of two-component systems involved in biofilm formation, such as *MSMEG_1875* (*mtrB*), *MSMEG_1874* (*mtrA*), *MSMEG_0246* (*prrB*), and *MSMEG_0244* (*prrA*) (Figure 6C). Δ*cnpB* showed porphyrin accumulation in the long-term medium (Figure 5 and Supplementary Figure 3). Elevated c-di-AMP in *M. smegmatis* significantly upregulated the expression of porphyrin-metabolizing enzymes in 3-day culture, including *MSMEG_2618* (uroporphyrin-III C-methyltransferase), *MSMEG_0954* (uroporphyrinogen-III synthase), and *MSMEG_2780* (uroporphyrinogen decarboxylase) (Figure 6C), and downregulated the expression of *MSMEG_4525* (putative oxygen-independent coproporphyrinogen III oxidase) (Figure 6C), which may result in more porphyrin production in Δ*cnpB*.

It has been reported that c-di-AMP specifically binds to K^+^ transport proteins such as KdpD and KtrA, TrkA, and TetR family proteins to inhibit potassium transporter function (Bai et al., 2014; Kim et al., 2015; Ali et al., 2017). We found that several potassium ion transport gene transcriptions were downregulated in Δ*cnpB*, including *MSMEG_5392* (*kdpA*), *MSMEG_5393* (*kdpB*), *MSMEG_5394* (*kdpC*), *MSMEG_5395* (*kdpD*), and *MSMEG_2769* (*trkB*) (Figure 6C). These data suggested that c-di-AMP inhibited the uptake of potassium ions by bacteria not only because of its direct binding to potassium ion transporters but also may be related to the inhibition of transporters transcription.

It has been demonstrated that c-di-AMP modulates bacterial virulence and affects host immune responses (Blander and Barbet, 2018). There were several different protein bands appearing in supernatants between wild-type and Δ*cnpB* strain but they were not obvious in bacterial lysates (Supplementary Figures 4A,B). qRT-PCR showed that elevated c-di-AMP downregulated the expression of early secreted antigens of *MSMEG_0066* (*esat-6, *esxA**) and *MSMEG_6398* (*ag85, *fbpA**) in Δ*cnpB* (Figure 6C), and Δ*cnpB* seemed to secrete less Ag85 than wild-type *M. smegmatis* (Supplementary Figure 4C). While host cell death-associated *MSMEG_1638* and PE/PPE family proteins of *MSMEG_5350* (*PPE 63*) were upregulated, it was speculated that CnpB deletion might change the immunogenicity of *M. smegmatis in vivo*.

### Δ*cnpB* Induced Similar Humoral and Cellular Immune Responses in Mice

**FIGURE 7:**
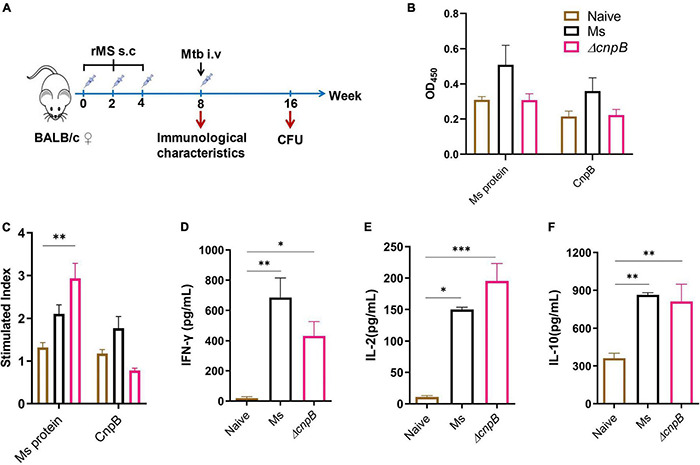
Immune responses induced by Δ*cnpB* in mice. **(A)** The immunization and *M. tuberculosis* infection strategy scheme. Briefly, 6- to 8-week-old female BALB/c mice were immunized subcutaneously (s.c) with PBS (naïve group) and 10^7^ CFU of wild-type *M. smegmatis* and Δ*cnpB* strains, respectively. Mice were boosted two times at 2-week intervals. Then, 4 weeks after the last immunization, mice were infected intravenously (i.v) with 5 × 10^4^ CFU of *M. tuberculosis* H37Ra. **(B)** Specific IgG levels against *M. smegmatis* protein and CnpB in sera were assayed by ELISA, and dilutions were 1:200 and 1:400, respectively. **(C)** Splenocyte proliferation of immunized mice stimulated by *M. smegmatis* protein extract (25 μg/ml) or CnpB (5 μg/ml) *in vitro*. **(D–F)** Splenocytes of mice were stimulated with *M. smegmatis* protein extract (25 μg/ml) for 72 h *in vitro*, and cytokine productions of IFN-γ **(D)**, IL-2 **(E)**, and IL-10 **(F)** in the supernatant were detected using ELISA. *p < 0.05, ^**^p < 0.01, ^***^p < 0.001.

c-di-AMP could induce innate immune responses and enhance antigen-induced Th1/Th2/Th17 responses as an adjuvant (Ebensen et al., 2011; Devaux et al., 2018; Ning et al., 2021). It has been revealed that *M. tuberculosis*Δ*cnpB* could trigger host innate immune responses, which could promote the clearance of bacteria (Yang et al., 2014; Dey et al., 2017). We demonstrated that recombinant BCG with elevated c-di-AMP by overexpressing DisA induced enhanced immune responses and provided similar protection as BCG in the same *M. tuberculosis* intravenous infected mice model (Ning et al., 2019). In this study, humoral and cellular immune responses induced by Δ*cnpB* were also investigated (Figure 7A). Δ*cnpB* subcutaneous immunization induced comparable IgG levels with the naïve group (Figure 7B). Splenocyte proliferation assay showed that Δ*cnpB* immunization induced significant proliferation than the naïve group responding to mycobacterial proteins (*p* < 0.01) but no more than that of *M. smegmatis* immunization (Figure 7C). Δ*cnpB* immunization induced increased Th1 (IFN-γ, IL-2) and Th2 (IL-10) cytokines’ secretion in supernatants of splenocytes restimulated with *M. smegmatis* proteins than control mice (*p* < 0.05), but no difference was found between two immunization groups (Figures 7D–F). These results showed that the elevated c-di-AMP level could not enhance the immunogenicity of *M. smegmatis* as immunized subcutaneously in mice.

### Δ*cnpB* Induces Enhanced Immune Responses After *M. tuberculosis* Infection in Mice

**FIGURE 8:**
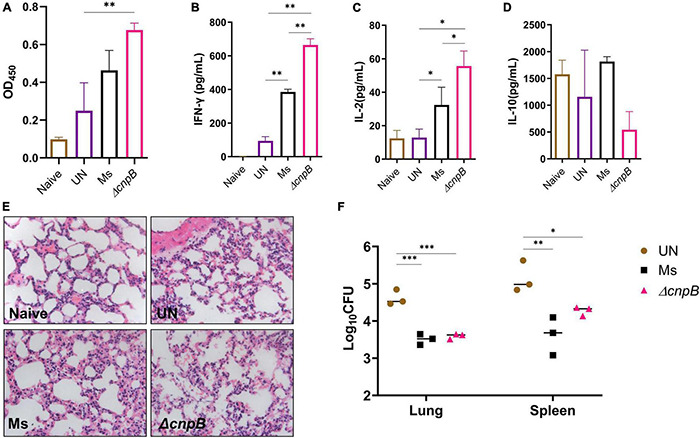
Immune responses induced by Δ*cnpB* post-*M. tuberculosis* infection and protection efficiency. **(A)** Specific IgG level against *M. tuberculosis* protein in sera (1:200) of immunized mice after *M. tuberculosis* H37Ra infection. **(B–D)** Splenocytes of mice were stimulated with H37Ra protein extracts (25 μg/ml) for 72 h *in vitro*, and cytokine productions of IFN-γ **(B)**, IL-2 **(C)**, and IL-10 **(D)** in the supernatant were detected using ELISA. **(E)** H&E-stained lung sections of each group of mice at 8 weeks post-*M. tuberculosis* infection. Gross pathology changes were observed by microscope (10 × 40). **(F)** At 8 weeks post-*M. tuberculosis* infection, bacterial burdens in the mice lungs and spleens were counted by plating on 7H10 plates. *p < 0.05, ^**^p < 0.01, ^***^p < 0.001.

Post 4 weeks of the last immunization, mice were challenged with *M. tuberculosis* intravenously (Figure 7A). Then, 8 weeks post-infection, Δ*cnpB* immunized mice showed an increase in IgG level, which were significantly higher than that of challenged mice without immunization (UN), and higher than the Ms group though no significant difference (Figure 8A). After *M. tuberculosis* infection, both wild-type and Δ*cnpB* strain-immunized mice produced significantly increased Th1 cytokines (IFN-γ, IL-2) rather than Th2 cytokine (IL-10) compared with unimmunized mice (Figures 8B–D). It was noted that Δ*cnpB* immunization could induce more IFN-γ and IL-2 secretion in splenocyte supernatant than that of wild-type *M. smegmatis* (Figures 8B,C). IL-10 secretion showed no differences between groups (Figure 8D). Overall, these results indicated that Δ*cnpB* immunization promoted significant Th1-type immune responses but not Th2-type immune responses after *M. tuberculosis* infection in mice.

### Δ*cnpB* Confers Protection Comparable to Wild-Type Strain Against *M. tuberculosis* Intravenous Infection

Δ*cnpB* immunization stimulated Th1 instead of Th2 immune responses, which is considered to induce immunopathological damage after *M. tuberculosis* infection in mice (Da Silva et al., 2015). At 8 weeks post-infection, all infected mice showed similar pathological manifestations of chronic inflammation by H&E staining, including partially damaged alveolar structures, occasional thickening of alveolar mesenchyme, inflammatory cell infiltration, and erythrocyte and histological fluid exudation, but no granuloma or nodules were found in the slide of lungs (Figure 8E).

After mice were infected with *M. tuberculosis* intravenously, both *M. smegmatis* strains’ immunization could reduce the bacteria burdens in both lungs (0.87–1.02 lgCFU) and spleens (0.87–1.3 lgCFU) compared to the unimmunized group (*p* < 0.05) (Figure 8F). There were no statistical differences in bacteria loads of lungs and spleens between *M. smegmatis* and Δ*cnpB*-immunized mice (Figure 8F). Overall, Δ*cnpB* immunization provided similar protections as wild-type *M. smegmatis* against *M. tuberculosis* intravenous infection in mice.

## Discussion

It has been reported that *cnpB* deletion did not cause a c-di-AMP level change in *M. smegmatis*, and overexpressing *cnpB* led to a drastic decrease in the c-di-AMP level (Tang et al., 2015). In this study, our results showed that *cnpB* deletion resulted in the accumulation of c-di-AMP at approximately 1.5-folds, and overexpressing *cnpB* led to decreased c-di-AMP level in *M. smegmatis* (Figure 1D). Thus, our results confirmed that CnpB as a phosphodiesterase handles c-di-AMP degradation, and the c-di-AMP level could be regulated by the *cnpB* expression level.

c-di-AMP plays a critical role in several physiological processes and is an indispensable molecule in *Bacillus thuringiensis* (Zheng et al., 2015). Increasing c-di-AMP levels in bacteria led to smaller colony sizes in *Streptomyces venezuelae* (Latoscha et al., 2020), as well as in *M. smegmatis* with DisA overexpression (Zhang and He, 2013; Tang et al., 2015). We found that Δ*cnpB* with elevated c-di-AMP level forms smaller, whitish, and moist colonies compared with wild-type strain (Figure 2A). Moreover, Δ*cnpB* showed shorter length (Figure 2D) and slower growth in 7H9 medium (Figure 3 and Supplementary Figure 2), which may be partially attributed to the smaller colonies, as well as reduced optical density (Figure 3 and Supplementary Figure 2). In our previous study, we found that Δ*cnpB* of *M. tuberculosis* caused ∼30% reduction in bacterial length (Yang et al., 2014) and a similar reduction in *disA*-overexpressing BCG (Ning et al., 2019), but did not find obvious changes in colony morphology.

Bacterial biofilms are often associated with chronic infections in human and contribute to the virulence and drug tolerance of *M. tuberculosis* (Chakraborty et al., 2021). It was reported that inhibition of DisA impedes *Enterococcus faecalis* biofilm formation (Chen et al., 2018). It was also determined that increased c-di-AMP level by deletion of phosphodiesterase A promoted biofilm formation in *Streptococcus mutans* (Peng et al., 2016). In this study, we reported that Δ*cnpB* with elevated c-di-AMP level inhibited biofilm formation (Figure 4). The qRT-PCR analysis identified that biofilm formation genes of two component systems (MtrA/B and PrrA/B) were downregulated and endoglucanase A(MSMEG_6752)was upregulated in Δ*cnpB*, which supported the observation of the biofilm formation reduction above in the elevated c-di-AMP level strain (Figure 6C, data not shown). Thus, c-di-AMP may regulate biofilm formation through distinct mechanics in different bacteria.

Many mycobacteria produce yellow, orange, or less frequently, salmon pink pigments either in the dark (scotochromogens) or upon exposure to light (photochromogens) (Provvedi et al., 2008; Stockel et al., 2015). These pigments have been characterized as carotenoids, a class of polyterpene lipids whose functions are to act as free radical scavengers and to protect the cells against photodynamic injuries (Provvedi et al., 2008). *MSMEG_5609* has been reported as carotenoid oxygenase and is regulated by *M. smegmatis MSMEG_1804* (*sigF*) (Provvedi et al., 2008). In this study, we found that Δ*cnpB* showed a whitish colony (Figure 2). However, RNA-seq analysis showed that no difference was found in the transcription of *MSMEG_5609* and *MSMEG_1804* between Δ*cnpB* and wild-type (data not shown). Thus, the role of c-di-AMP in regulating colony morphology and coloration of mycobacterium remains unknown and needs to be investigated.

Surprisingly, we found that Δ*cnpB* produced more brown pigment in long-term culture (Figure 5A). By analyzing solubility and properties, fluorescence analysis, full-wavelength scanning, and spectral analysis, the brown pigment accumulation in Δ*cnpB* supernatant was proved to be a water-soluble porphyrin (Figure 5; Gouterman, 1959, 1961; Dolphin, 1978; Nikitushkin et al., 2016), which was supported by the upregulated porphyrin synthesis-associated genes in Δ*cnpB* by RNA-seq analysis and qRT-PCR (Figure 6B,C). Ferrous ions could be directly incorporated into various porphyrins for the storage of iron within the bacterial cytoplasm (Ratledge, 2004). The overall process of iron acquisition and its utilization is under very genetic tight control, which contributes to the virulence of mycobacteria as well as the development of tuberculosis (Ratledge, 2004). We detected the iron concentration and found an increase of iron in Δ*cnpB*::C supernatant of 60-week culture (data not shown), suggesting that c-di-AMP may regulate porphyrin as well as iron metabolism in mycobacterium.

It was reported that the c-di-AMP level was tightly controlled in *Bacillus subtilis*, and the accumulation of c-di-AMP impaired the growth of *B. subtilis*, which can be partially suppressed by elevated concentrations of magnesium (Mehne et al., 2013), whereas the lack of c-di-AMP also was detrimental to *B. subtilis* cell growth (Mehne et al., 2013). In this study, most phenotypes of *cnpB*-overexpressed strains were similar to those of the wild-type *M. smegmatis*. It was found that *disA*-overexpressed strain of BCG showed elevated c-di-AMP and shorter length than Δ*cnpB* of *M. smegmatis* but did not find changes in growth and colony morphology (Ning et al., 2019). Thus, we speculated that low c-di-AMP by *cnpB* overexpression may also be regulated by an unknown compensatory pathway with the unknown mechanism, which will be investigated in our further study.

An increasing number of reports have proved that c-di-AMP regulates diverse bacterial physiological processes (Yin et al., 2020; Yinlan Bai, 2020), as well as host immune responses (Devaux et al., 2018). It was also reported that Δ*cnpB* of *M. tuberculosis* was easier to be cleared from the host, which may be the result of a combination of enhanced immune response and strain virulence changes (Yang et al., 2014; Dey et al., 2017). Further, we detected the immunogenicity of Δ*cnpB*. In fact, Δ*cnpB* induced similar levels of humoral and cellular immune responses as *M. smegmatis* did, except significant proliferation of splenocytes against *M. smegmatis* proteins (Figure 7). Unexpectedly, Δ*cnpB* induced significant humoral immune responses compared with unimmunized (UN) mice. Moreover, Δ*cnpB*-immunized mice could produce more Th1 cytokines of IFN-γ and IL-2 rather than Th2 cytokine (IL-10) after *M. tuberculosis* infection (Figure 8).

Antigen-specific Th1 response is usually considered a marker of protective immune responses against *M. tuberculosis* infection (Zeng et al., 2018). Compared with wild-type *M. smegmatis*, it was found that Δ*cnpB* immunization could induce enhanced Th1 cellular immune responses after *M. tuberculosis* infection, which could promote the elimination of *M. tuberculosis* infection. Reports including ours have found that *M. smegmatis* could be used as a live vaccine vector or a therapeutic vaccine (Junqueira-Kipnis et al., 2013; Alves Da Silva et al., 2014; Wang et al., 2014; Liu et al., 2015). Previously, our and other studies have demonstrated that *cnpB*-deficient *M. tuberculosis* exhibited attenuated virulence (Yang et al., 2014; Dey et al., 2017). Therefore, the safety of *M. smegmatis* Δ*cnpB* is better in theory than wild tyoe strain, coupled with the stronger immune response induced by Δ*cnpB*, and it may replace *M. smegmatis* as a vaccine or vaccine carrier. However, elevated c-di-AMP level did not increase the protective efficiency of *M. smegmatis* against *M. tuberculosis* venous infection (Figure 8). Previously, we demonstrated that a recombinant BCG with the elevated c-di-AMP level by overexpressing DisA provided similar protection as BCG in the same mouse model of *M. tuberculosis* intravenous infection (Ning et al., 2019). It was reported that the same recombinant BCG provided improved protection after aerosol infection of *M. tuberculosis* in guinea pigs (Dey et al., 2020). Thus, Δ*cnpB* should be further evaluated for the protection with the aerosol challenge of *M. tuberculosis*, using more susceptible animals such as guinea pigs.

Taken together, our results provided more evidence that elevated c-di-AMP could affect colony morphology, bacterial length, growth, and potassium transporters of *M. smegmatis*, consistent with previous research in several bacteria, including other mycobacteria (Corrigan et al., 2011; Du et al., 2014; Chen et al., 2018). Moreover, we reported that elevated c-di-AMP could inhibit biofilm formation and induce porphyrin accumulation, which may affect bacterial drug resistance and virulence, by regulating their associated gene expressions in *M. smegmatis*. Compared with wild-type *M. smegmatis*, *cnpB*-deleted strain with elevated c-di-AMP level could induce enhanced immune responses, especially protective Th1 immune responses after *M. tuberculosis* intravenous infection in the mouse model, which suggested *M. smegmatis* with the elevated c-di-AMP level as a vaccine against tuberculosis.

## Data Availability Statement

The bulk RNA-seq datasets presented in this study can be found in online repositories. The names of the repository/repositories and accession number(s) can be found below: NCBI BioProject—PRJNA808387.

## Ethics Statement

The animal study was reviewed and approved by Institutional Animal Ethics Center of Air Force Medical University.

## Author Contributions

HN, XL, and YX performed the most experiments and analyzed the data. LB, WZ, and LW conducted some animal experiments. JK and YL conducted several immunological assays. YM provided guidance in the study of bacterial physiology. HN and YB wrote the manuscript. YB and GB conceived and designed the research. YB supervised the work. All authors have read and agreed with the data.

## Conflict of Interest

The authors declare that the research was conducted in the absence of any commercial or financial relationships that could be construed as a potential conflict of interest.

## Publisher’s Note

All claims expressed in this article are solely those of the authors and do not necessarily represent those of their affiliated organizations, or those of the publisher, the editors and the reviewers. Any product that may be evaluated in this article, or claim that may be made by its manufacturer, is not guaranteed or endorsed by the publisher.
