# Supplementary material for: c-di-AMP Accumulation Regulates Growth, Metabolism, and Immunogenicity of Mycobacterium smegmatis
Source: Front Microbiol. 2022 May 24;13:865045. doi: 10.3389/fmicb.2022.865045 (PMC9171234; doi:10.3389/fmicb.2022.865045)
Supplement: Supplementary file 1 [file Data_Sheet_1.docx]

**Table S1 Strains, plasmids, and antibodies used in this study**

| **Strain and Plasmid** | **Feature** | **Source** |
| --- | --- | --- |
| **Mycobacteria** |  |  |
| *M. smegmatis* mc^2^-155 | Wild type strain of *M. smegmatis* | From National Institutes for Food and Drug Control of China |
| Δ*cnpB* | *cnpB* deletion mutant strain | This study |
| Δ*cnpB*::C | *cnpB* complementary strain | This study |
| Δ*cnpB*::O | *cnpB* overexpressed strain | This study |
| *M. tuberculosis* H37Ra | Wild type of *M. tuberculosis* attenuated strain | National Institutes for Food and Drug Control of China |
| ***E. coli*** |  |  |
| DH5α  BL21 | Plasmid construction  Expression of protein | This study  This study |
| **Plasmids** |  |  |
| pJV53 | Expressing homologous recombination enzymes of gp60 and gp61, Kan^R^ | Addgene |
| pMSG360zeo | *loxp*-*zeo*-*loxp* | Addgene |
| PW75 | Recombinant pMSG360zeo carrying *cnpB* upstream and downstream fragments, Zeo^R^ | This study |
| PW86 | Mycobacterium-*E. coli* *cnpB* single copy shuttle expression vector, Hyg^R^ | This study |
| PW87 | Mycobacterium-*E. coli* *cnpB* multiple copy shuttle expression vector, Hyg^R^ | This study |
| PW92 | CnpB prokaryotic expression vector of pET28a(+)-*cnpB*, Kan^R^ | This study |
| **Antibodies** |  |  |
| anti-CnpB pAb | Mouse polyclonal antibody to CnpB (MSMEG_2630) | Our lab |
| anti-Ag85 pAb | Mouse polyclonal antibody to Ag85 (MSMEG_6398), | Our lab |
| anti-PdxH pAb | Mouse polyclonal antibody to PdxH (MSMEG_5675) | Our lab |

**Table S2 Primers used in this study**

| **Oligo sequence (5’ to 3’)** *^a^* | **Description** |
| --- | --- |
| **Strains construction and verification** |  |
| tttgatatcggccatcgagtacgagatc | *cnpB* upstream for knockout, forward |
| gcgaagcttggtcttgggatcggttgtcgtc | *cnpB* upstream for knockout, reverse |
| ttttctagagcgggatactccgcgacc | *cnpB* downstream for knockout, forward |
| gcgggtaccgaaagcatcggcaccgcaac | *cnpB* downstream for knockout, reverse |
| aactacatcgacccttccgc | *cnpB* internal, forward |
| gtgcatcggttcgatctcct | *cnpB* internal, reverse |
| gtgatctcggtgagccactc | *MSMEG_6080* internal, forward |
| aagtccagcagttcgctgtc | *MSMEG_6080* internal, reverse |
| tttggtaccatgccggtgacgacaacc | *cnpB* complementation, forward |
| tttggatcctcagccaagggcccgtgc | *cnpB* complementation, reverse |
| tttaagcttatgccggtgacgacaacc | *cnpB* overexpression, forward |
| tttaagctttcagccaagggcccgtgc | *cnpB* overexpression, reverse |
| **qRT-PCR** |  |
| cgacgacctcgactccgacgac | *MSMEG_2758* (*sigA*), forward |
| gccttcctggatgaggtcgagga | *MSMEG_2758* (*sigA*), reverse |
| agctgtacctgatcttcccg | *MSMEG_1875 (mtrB)*, forward |
| ctcttcgagctgctggatct | *MSMEG_1875 (mtrB)*, reverse |
| ggagccgacgactatgtgat | *MSMEG_1874 (mtra)*, forward |
| gtatccccacacctgttcga | *MSMEG_1874 (mtra)*, reverse |
| gatgcggcacaactcgatc | *MSMEG_0246 (prrB)*, forward |
| cgctcaacaagatggagtcg | *MSMEG_0246 (prrB)*, reverse |
| tacctgaccaaaccgttcga | *MSMEG_0244 (prrA)*, forward |
| aagtcgtagccccacacc | *MSMEG_0244 (prrA)*, reverse |
| cggctacgaagaggtgctc | *MSMEG_2618*, forward |
| tgcatcccaattcactaacg | *MSMEG_2618*,reverse |
| cctacgacgaggttttcgac | *MSMEG_0954*, forward |
| ccaccggtcttgatcatctc | *MSMEG_0954*, reverse |
| ccatcctgttctccgacatc | *MSMEG_2780*, forward |
| caccaggtaggaggccaac | *MSMEG_2780*, reverse |
| aggtcaccacagaagcgaac | *MSMEG_4525*, forward |
| agatgaggtcgaggttgacg | *MSMEG_4525*, reverse |
| gattccctggactggctgg | *MSMEG_1638*, forward |
| tagccgttctccatctcgac | *MSMEG_1638*, reverse |
| ctactacaaccccggcatga | *MSMEG_0234 (zmp1)*, forward |
| tacttggcaccctgatcgtc | *MSMEG_0234 (zmp1)*, reverse |
| acagaacaggtatgg | *MSMEG_0066 (esat-6)*, forward |
| tcaggcaaacattcc | *MSMEG_0066 (esat-6)*, reverse |
| catctgggtgtactgcggta | *MSMEG_6398(ag85)*, forward |
| ctctgcgggaagttgaacac | *MSMEG_6398(ag85)*, reverse |
| aagaatctgcccctcacaca | *MSMEG_5350 (PPE 63)*, forward |
| acgtccttggtttccgtact | *MSMEG_5350 (PPE 63)*, reverse |
| accaccatgagcacctatca | *MSMEG_0619 (PPE4)*, forward |
| gtcatgaagtccgtcagcag | *MSMEG_0619 (PPE4)*, reverse |
| tacagaacttcgtctcggcc | *MSMEG_5392 (kdpA)*, forward |
| ggtgcccagttctttgatgg | *MSMEG_5392 (kdpA)*, reverse |
| agtacttcgcgatcatcccg | *MSMEG_5393 (kdpB)*, forward |
| cgatgagtttgatgccgagg | *MSMEG_5393 (kdpB)*, reverse |
| aaggtgtacgggtcgagtg | *MSMEG_5394 (kdpC)*, forward |
| caccgagaaatcccagcatg | *MSMEG_5394 (kdpC)*, reverse |
| gccatcagatgtttcacgca | *MSMEG_5395 (kdpD)*, forward |
| tacagaacttcgtctcggcc | *MSMEG_5395 (kdpD)*, reverse |
| cctttggcatggacgagttc | *MSMEG_5396 (kdpE)*, forward |
| cgcgtaaatagtgggtctcg | *MSMEG_5396 (kdpE)*, reverse |
| gagaccgagaccaccaagtg | *MSMEG_2771 (trkA)*, forward |
| tcattcggaatccaggtcgt | *MSMEG_2771 (trkA)*, reverse |
| gcgcaacgaatggcttttc | *MSMEG_2769 (trkB)*, forward |
| cagttcgtcaccaccttcga | *MSMEG_2769 (trkB)*, reverse |
| *a* Underlined oligo DNA is restriction site. | |


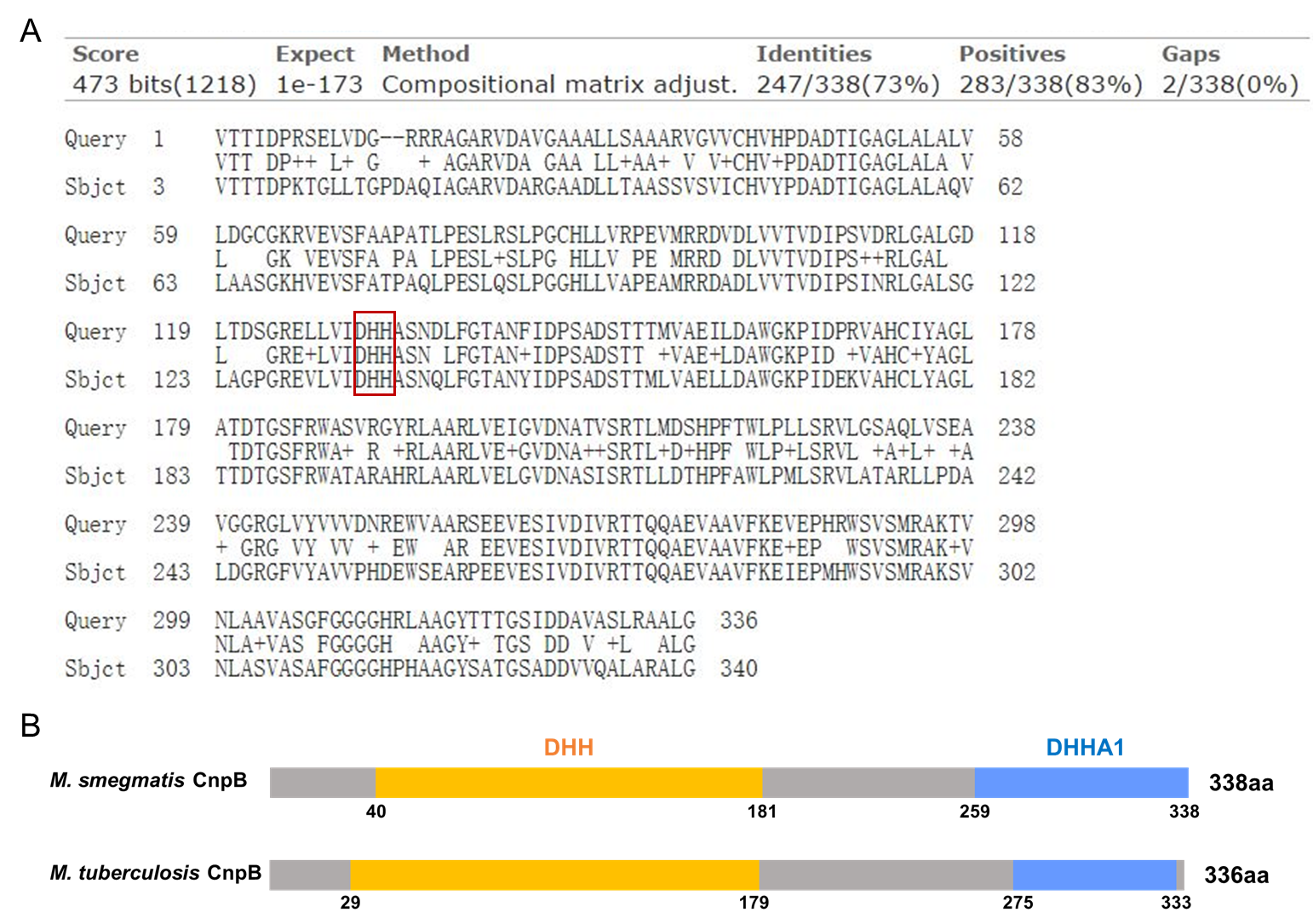


**Figure S1 CnpB amino acid sequences alignment between *M. tuberculosis* and *M. smegmatis*.** (A) Alignment of *M. tuberculosis* CnpB (Query) and *M. smegmatis* CnpB (Sbjct) sequences. The amino acids shown in the red rectangle is DHH motif. (B) Amino acid sites of DHH and DHHA1 domains of CnpB in *M. tuberculosis* and *M. smegmatis*, respectively.


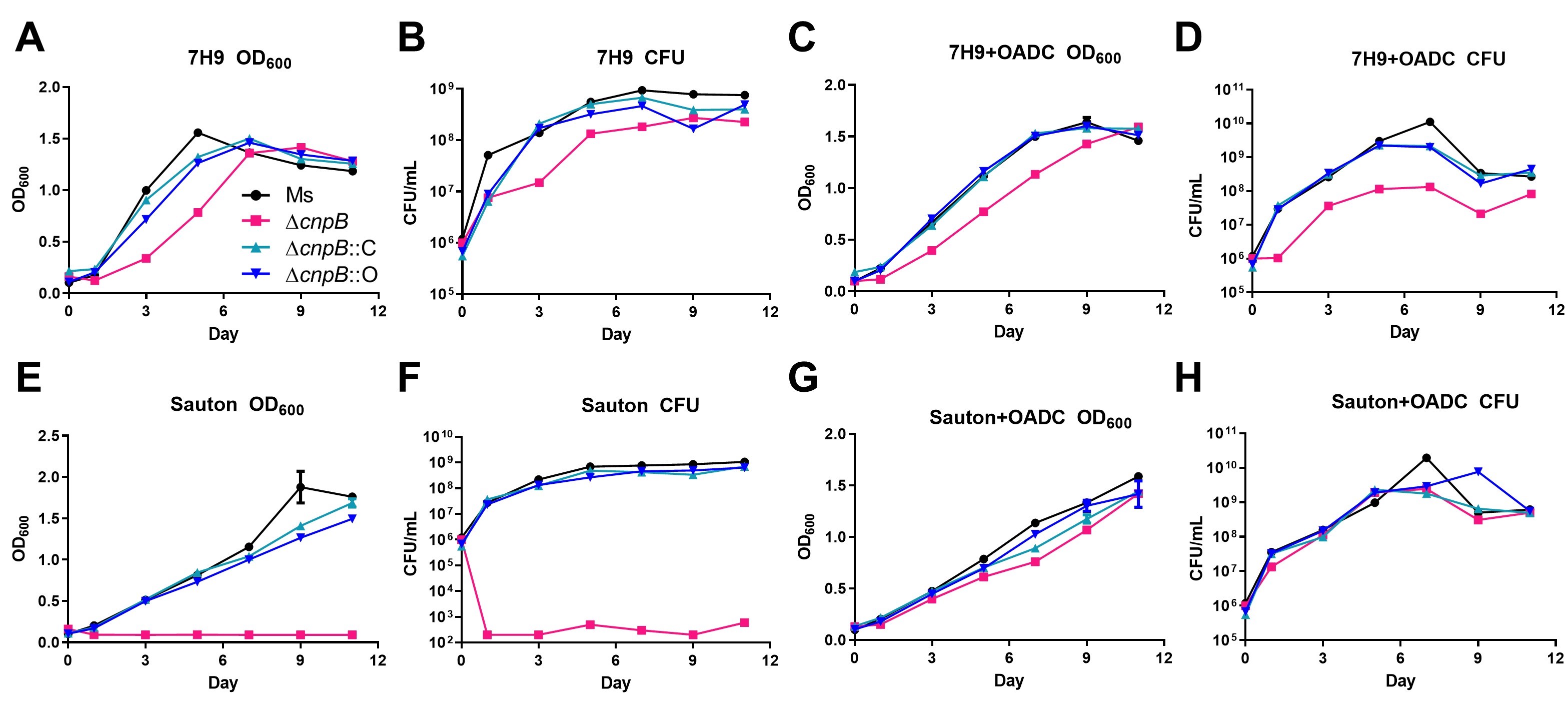


**Figure S2** **Detection of bacteria growth in broth liquid media (80rpm).** Each strain was inoculated at 2.5×10^6^ CFU/mL in media of 7H9 (A), 7H9+OADC (C), Sauton’s (E), Sauton’s +OADC (G). Bacteria were monitored at OD_600_ (A, C, E, G) and corresponding CFUs (B, D, F, H) numeration at indicated time points, respectively. The growth curves were generated according to three repeats.


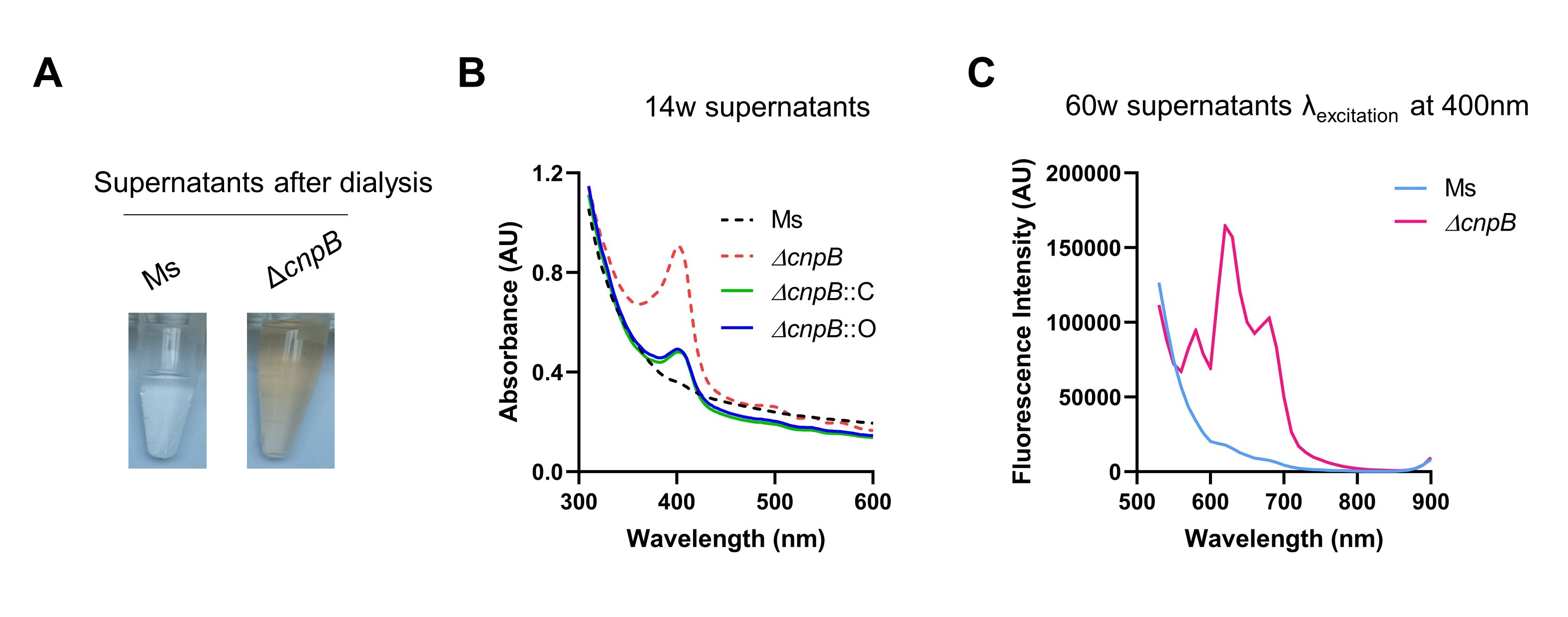


**Figure S3** ***ΔcnpB* produceds more brown pigment during the long-term static culture.** (A) Observation of 14w supernatants after dialysis. (B) Absorption spectra of 14-week supernatants was recorded by full-wavelength (100 - 1 000 nm) scanning. (C) Fluorescence measurements of 60w supernatants were analyzed at the excitation wavelength (λ_excitation_) 400 nm.

**
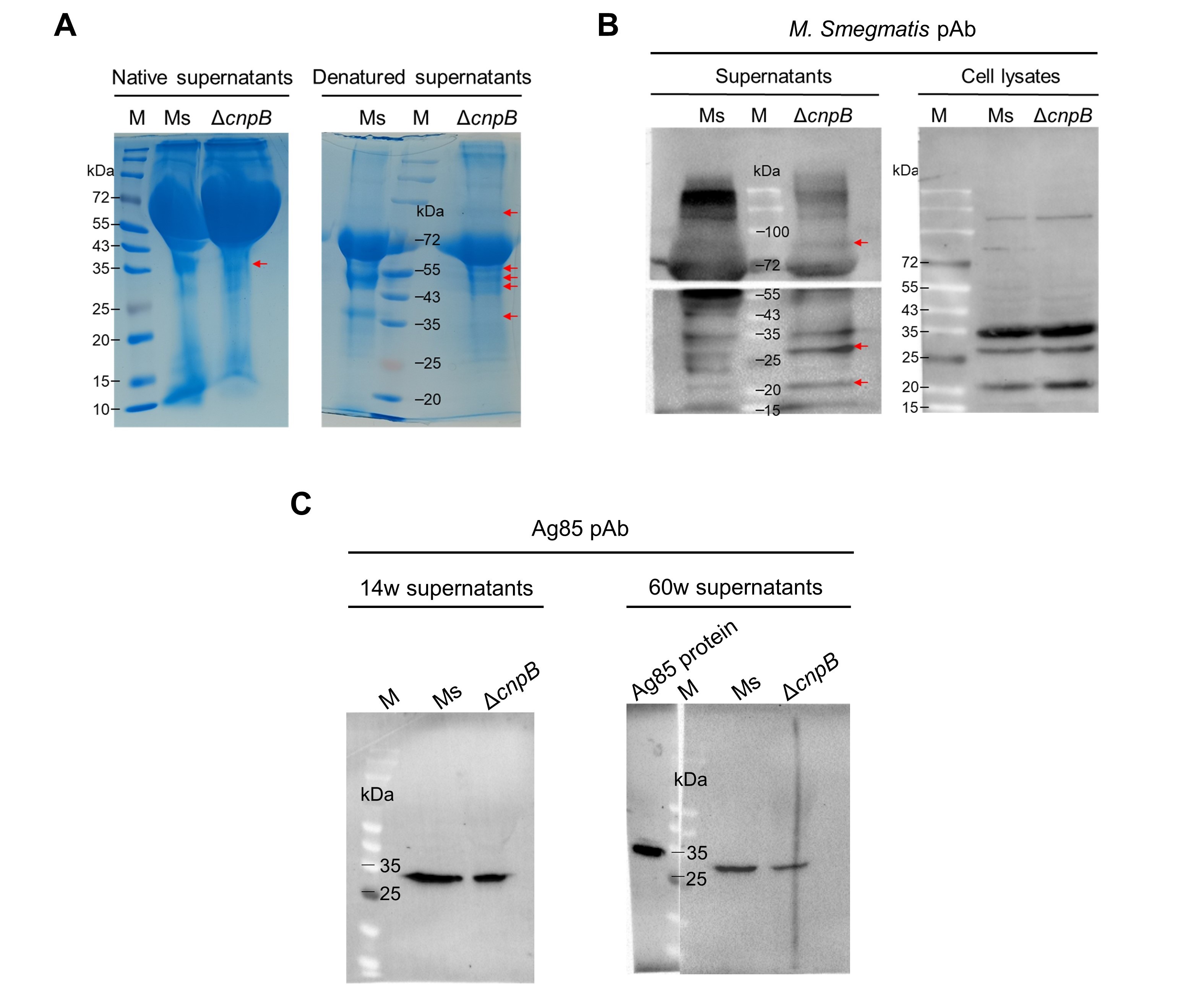
**

**Figure S4**  ***ΔcnpB* produce** (A) After 14-week of stationary culture, native and boil-denatured supernatants were analyzed by SDS-PAGE. The red arrows indicated possible new protein bands of *ΔcnpB*. (B) After 14-week of stationary culture, supernatants were condensed by saturated ammonium sulfate. Condensed-supernatants and bacteria lysates were analyzed by Western blot using *M. smegmatis* polyclonal antibody. The red arrows indicated the *M. smegmatis*-specific differential protein bands in *ΔcnpB* supernatant after 14-week culture. (C) Western blot analysis of Ag85 secretion levels in concentrated-supernatants at 14 and 60 weeks.
